# A National audit of the care of patients with acute kidney injury in England and Wales in 2019 and the association with patient outcomes

**DOI:** 10.1016/j.clinme.2024.100028

**Published:** 2024-02-20

**Authors:** M.P.M. Graham-Brown, A. Casula, M. Savino, T. Humphrey, R. Pyart, M. Amaran, J. Williams, K. Crowe, J.F. Medcalf, NEPHwork consortium collaborators. All involved in data collection and or local project setup and supervision, Dr Keegan Lee, Dan Cooper, Dr Edward Carr, Dr Amar Marthi, Dr Oscar Swift, Dr Katherine Hull, Dr Ailish Nimmo, Dr Hui Liewm, Dr Behram Tariq, Dr Jenny Whitehead, Dr Naomi Edney, Dr Daniel Whitbread, Dr Maha Mohamed, Dr Sam Duffy, Dr Gwenno Edwards, Dr Rachael Czajka, Dr Syed Hasan Ahmad, Dr Jennifer Joslin, Dr Esther Siaw Tsin Yong, Dr Saurabh Chaudry, Dr Daniel McGuinness, Dr Sarah Defreitas, Dr Heba Nosseir, Dr Katherine Seal, Dr Mo Amaran, Dr Kavita Gulati, Dr Muhammad J Azam, Dr Jennifer Williams, Smith-Jackson, Dr Bo-song Yin, Dr Rishana Shuaib, Dr Mosammat Akter, Dr Ryoki Arimoto, Dr Rotimi Oluyombo, Dr Mark Davies, Dr Purvi Patel, Tony Best-Trent, Dr Heidy Handra, Sarah Mackie, Kelly Wright, Dr Manzur Rahman, Dr Hashem Cheema, Dr Abbas Sardar, Dr Lucy Harvard, Dr Matthew Brook, Dr Emma Elphic, Dr Mawahib Ahmed, Dr Kanbar Ammar, Dr Madi Harbe, Dr Emma Corke, Dr Hannah Stacey, Dr Mosab Yousif, Dr Dalal Mohamed, Dr Lae Thandar Soe, Dr Adenwalla Sherna, Dr Lauren Soutter, Dr Maria Davari, Dr Sneha Abburu, Dr James Wells, Dr Claire Winterbottom, Dr Matt Bottomley, Dr Holly Morris, Dr Anavami Sadiq, Dr Sajeda Youssouf

**Affiliations:** 1 St George's University Hospitals NHS Foundation Trust; 2 Cambridge University Hospitals NHS Foundation Trust; 3 East and North Hertfordshire NHS Foundation Trust; 4 University Hospitals of Leicester NHS Foundation Trust; 5 North Bristol NHS Foundation Trust; 6 South Tees Hospitals NHS Foundation Trust; 7 Royal Devon and Exeter Hospital NHS Foundation Trust; 8 Newcastle Upon Tyne Hospital NHS Foundation Trust; 9 South Tyneside and Sunderland NHS Foundation Trust; 10 Cardiff & Vale University Health Board; 11 Bradford Teaching Hospitals NHS Foundation Trust; 12 East Suffolk North Essex NHS Foundation Trust; 13 King's College Hospital NHS Foundation Trust; 14 Western Sussex University Hospitals NHS Foundation Trust; 15 Barts Health NHS Trust; 16 Barts Heath NHS Trust; 17 Brighton & Sussex University Hospitals NHS Foundation Trust; 18 Brighton & Sussex University Hospitals NHS Foundation; 19 Epsom and St Helier NHS Foundation Trust; 20 Imperial College Healthcare NHS Trust; 21 University Hospitals of North Midlands NHS Trust; 22 University Hospital Plymouth NHS Trust; 23 Royal Wolverhampton NHS Trust; 24 Royal Berkshire Hospital NHS Trust; 25 Norfolk and Norwich University Hospitals NHS Foundation Trust; 26 Betsi Cadwalader University Health Board; 27 Royal Berkshire Hospital NHS Foundation Trust; 28 University Hospitals Plymouth NHS Trust; 29 Oxford University Hospitals NHS Foundation Trust; 30 Shrewsbury and Telford Hospital NHS Trust; a Department of Cardiovascular Sciences, University of Leicester, LE1 9HN, United Kingdom; b NIHR Leicester Biomedical Research Centre, University Hospitals of Leicester NHS Trust, Leicester, United Kingdom; c John Walls Renal Unit, Leicester General Hospital, University Hospitals of Leicester NHS Trust, United Kingdom; d UK Renal Registry, United Kingdom; e Bristol Royal Infirmary, Division of Acute Medicine; f Department of Renal Medicine, Cambridge University Hospitals NHS Foundation Trust, United Kingdom; g Renal Unit, St George's Hospital, London, United Kingdom; h School of Medicine, University of Exeter, United Kingdom; i Glasgow Renal & Transplant Unit, NHS Greater Glasgow & Clyde, United Kingdom

**Keywords:** AKI, Acute Kidney Injury

## Abstract

**Background:**

Acute kidney injury (AKI) is a common complication of hospitalisations. This national audit assessed the care received by patients with AKI in hospital Trusts in England and Wales.

**Methods:**

Twenty four hospital Trusts across England and Wales took part. Patients with AKI stage2/3 were identified using the UK Renal Registry AKI master patient index. Data was returned through a secure portal with linkage to hospital episode statistic mortality and hospitalisation data. Completion rates of AKI care standards and regional variations in care were established.

**Results:**

989 AKI episodes were included in the analyses. In-hospital 30-day mortality was 31-33.1% (AKI 2/3). Standard AKI interventions were completed in >80% of episodes. Significant inter-hospital variation remained in attainment of AKI care standards after adjustment for age and sex. Recording of urinalysis (41.9%) and timely imaging (37.2%) were low. Information on discharge summaries relating to medication changes/re-commencement and follow-up blood tests associated with reduced mortality. No quality indicators relating to clinical management associated with mortality. Better communication on discharge summaries associated with reduced mortality.

**Conclusions:**

Outcomes for patients with AKI in hospital remain poor. Regional variation in care exists. Work is needed to assess whether improving and standardising care improves patient outcomes.

## Introduction

Acute kidney injury (AKI) is caused by a heterogenous group of conditions that result in a sudden decline in kidney function. It is common affecting 12,300 per million population in England in 2020^1^^,^^2^ and associates with 100,000 deaths each year across UK hospitals.^3^ Acute kidney injury affects one in five emergency hospital admissions and associates with a substantial increase in short-term morbidity and mortality, increased likelihood of development of chronic kidney disease (CKD) and increased healthcare utilisation costs, estimated to be £400–600 million per year.^4^^,^^5^

In 2009 the National Confidential Enquiry into Patient Outcome and Death (NCEPOD) report Acute Kidney Injury: Adding Insult to Injury, provided the impetus for concerted national efforts to improve the care delivered to patients with AKI.^3^ The report concluded that for patients who died in hospital with AKI, in 15% of cases the episode of AKI could have been prevented and patients only received a standard of care that was considered good in 50% of cases. Since publication of the report national efforts have prioritised projects to improve the care delivered to patients with AKI. Since 2014, NHS England mandated AKI alerts be incorporated into all testing laboratories in England to improve AKI detection and patient outcomes. More recently, these AKI alerts have been routinely returned from acute Trusts across England to the UK Renal Registry (UKRR) with patient demographic data to create the UKRR AKI master patient index (MPI), with subsequent linkage to hospital episode statistic (HES) data to allow Nationwide analyses of AKI care.^6^ This allowed publication of the first UKRR AKI report in 2019, based on analysis of alerts sent to the UKRR throughout 2018, which included submission of alerts from 87% of laboratories in England and over 500,000 AKI episodes from 1.5 million AKI alerts.^7^ This report showed 30-day mortality following hospital acquired AKI remained high (24%) and disproportionately affected those from lower socio-economic background.

Established in 2020, NEPHwork is a national network developed by UK renal trainees to develop and deliver nationally prioritised, audit, quality improvement and research projects.^8^ Supported by the UK Kidney Association, the UKRR and Kidney Research UK, the first NEPHwork project audited the care of patients with AKI admitted to hospital in England and Wales 10 years on from the original NCEPOD report. This report describes the care delivered to patients with AKI in hospitals across England and Wales, regional variations in care between centres and putative links between care quality audit standards and patient mortality.

## Methods

The audit was carried out across 24 acute NHS Trusts in England and Wales by 57 UK renal trainees and junior doctors. This was a retrospective case note audit completed between 1 December 2020 and 28 February 2021. The audit was registered in each participating Trust. Audit standards were agreed by a steering group based on NICE recommendations for emergency and acute medical care and on the 2019 UK Kidney Association AKI clinical practice guidelines.^9^^,^^10^

### AKI episode identification and audit processes

Acute kidney injury episodes from England were identified using the UKRR AKI-MPI. In 2015 NHS England mandated that all laboratories in England issue e-alerts to define episodes of AKI using the same algorithm.^11^ The algorithm classifies AKI as stage 1, 2 or 3 in keeping with the Kidney Disease: Improving Global Outcomes (KDIGO) AKI staging classification.^12^ These e-alerts are returned to the UKRR along with patient demographic information to create the UKRR AKI-MPI which is routinely linked to England HES data to identify admitted patients and also provide coded comorbidity. For this audit, all episodes of AKI stage 2 and 3 at participating sites in England between December 2018 and February 2019 were identified centrally from the UKRR AKI-MPI. Wales has a separate linked register of hospitalised AKI which was used to identify the patients in Welsh centres but not other coded problems. Scottish centres were not included in this audit as we could not establish robust data linkage within the timeframe.

Individual episodes for audit were sent through a secure data portal on the electronic AKI audit proforma (pre-populated with patient details and the episode of AKI for audit) to individuals completing the audit at participating Trusts. Individuals were given secure logins to access the data portal to complete the audit of patients at their Trusts. AKI episodes were defined as one or more e-alerts separated by no more the 30 days. Trainees locally completed the audit against electronic and paper notes and returned the proforma securely through the data portal. Audit data were checked for completeness by project managers at the UKRR and queries were returned to trainees locally to review and complete. The project aimed to audit 1,000 AKI care episodes to be of similar size to the NCEPOD report from 2009.

### Additional data collection, definitions and outcomes

Demographic data were collected from several sources. Patient age and gender were obtained from the UKRR AKI-MPI. The UKRR AKI-MPI also provided the residence postcode for patients from England, while for Welsh patients these were obtained through tracing from the NHS Batch Demographics Service. Postcodes were used to assign patients the Index of Multiple Deprivation (IMD),^13^ a measure of relative deprivation for small areas, categorised into quintiles for analysis (from 1=most deprived to 5=least deprived group). Ethnicity was acquired using the HES linkage for English patients and was acquired as an extra variable for completion during the audit for Welsh patients during audit data collection. Hospital admissions were classed as elective or emergency admissions based on the admission-method available from HES-linkage, and divided into admission with hospital-acquired AKI (HA-admissions) or community-acquired AKI (CA-admissions) based on time between first AKI-alert and hospital admission (HA- if AKI commencing from day 3 of a hospital admission, CA- if AKI beginning outside of hospital, or within the first 2 days of admission). The NHS Batch Demographics Service was used to trace dates of death for all patients at 30 days, 90 days and 1 year. Patient co-morbidity score was calculated using clinical coding from the current admission along with other admissions during the previous 12 months as previously demonstrated.^14^ It was necessary to exclude patients admitted to Welsh hospitals from analyses relating to subsequent hospital admission rates as these data were not available.

### Statistical analyses

Statistical analyses were performed using SAS version 9.4. Normality was assessed using histograms and Shapiro-Wilk tests for continuous data. Normally distributed data are expressed as mean ± standard deviation (SD). Non-normally distributed data are expressed as median and interquartile range (IQR). Categorical data are reported as frequency (%) of observation. Audit measures and demographic characteristics are presented for the full cohort and by peak AKI stage. Variation in quality indicators between Trusts are represented with funnel plots, with attainment adjusted for age and sex with logistic analysis. Mortality outcomes are summarised for the full cohort and by AKI stage, with variation between Trusts adjusted for age and sex using logistic analyses and presented as a funnel plot. Relationships between attainment of audit care quality indicators and mortality were assessed with univariate logistic regression adjusted by age for odds ratio of mortality. Care quality indicators shown to be significant predictors of outcome on univariate analysis were assessed using multivariable logistic regression models adjusted by baseline covariates to assess independence of association with mortality outcomes. Adjustment factors considered for inclusion in the full model are age, gender, ethnicity, baseline renal function, IMD, cause of AKI, AKI stage, admission type and comorbidity score. A *p*-value of <0.05 was considered significant for associations between mortality and attainment of care quality indicators.

## Results

**Fig. 1:**
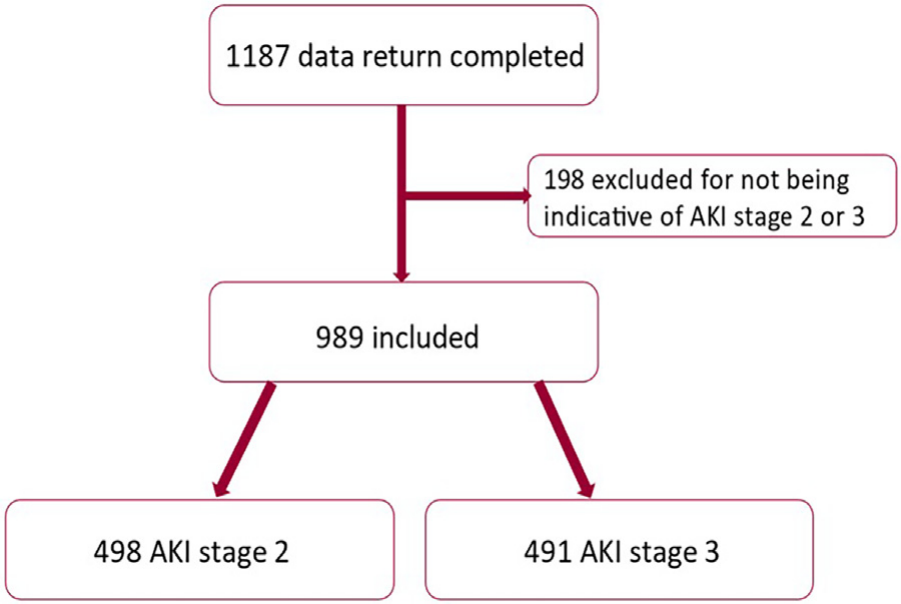
Data return and AKI stage for study cohort.

The NEPHwork AKI national audit was attended by 57 registrars from 24 NHS acute Trusts, 22 in England and 2 in Wales. The audit covered all geographical regions of England, apart from the Northwest. A total of 1,187 AKI episodes were evaluated and returned. Of these, 989 were included in the study cohort for analysis (Fig. 1). The sociodemographic and clinical features of AKI episodes are shown in Table 1. Most episodes of AKI occurred in patients over the age of 65, 45.9% were female and the majority of patients in this audit were from a white ethnicity background (90.2%). Sepsis and hypovolaemia were the most common causes of AKI. Most cases of AKI developed from emergency admissions (92.8%) and most AKI cases were community acquired (72.1%). Renal function was only judged to have returned to baseline at 30 days in 50.1% of patients, and 2.4% of patients entered a maintenance dialysis programme. In-hospital and 30-day mortality were 31% and 33.1% respectively. Including all stages of AKI, there were 327 deaths at 30 days, 388 deaths at 90 days and 487 deaths at 1 year.

**Table 1:** Sociodemographic and clinical features of AKI episodes by peak stage AKI and for all episodes of AKI.

Variables	AKI stage 2	AKI stage 3	All AKI (AKI stage 2 and 3)
Total N (%)	498 (50.3)	491 (49.6)	989 (100.0)
*Age group (years) N (%)*			
18–29	10 (2.0)	7 (1.4)	17 (1.7)
30–49	31 (6.2)	46 (9.4)	77 (7.8)
50–64	72 (14.5)	96 (19.6)	168 (16.9)
65–74	109 (21.9)	99 (20.2)	208 (21.0)
75–84	157 (31.5)	141 (28.7)	298 (30.1)
≥85	119 (23.9)	102 (20.8)	221 (22.3)
Sex N (%)			
Female	245 (49.2)	209 (42.6)	454 (45.9)
Male	253 (50.8)	282 (57.4)	535 (54.1)
*Index of Multiple Deprivation quintile N (%) – missing N=2*			
Deprivation score-1	101 (20.3)	85 (17.3)	186 (18.8)
Deprivation score-2	115 (23.1)	102 (20.8)	217 (22.0)
Deprivation score-3	98 (19.7)	104 (21.2)	202 (20.5)
Deprivation score-4	99 (19.9)	89 (18.2)	188 (19.1)
Deprivation score-5	84 (16.9)	110 (22.4)	194 (19.7)
*Ethnicity N (%) – missing N=72*			
Asian	18 (3.8)	14 (3.1)	32 (3.5)
Black	16 (3.4)	20 (4.5)	36 (3.9)
Other/Mixed	11 (2.4)	11 (2.4)	22 (2.4)
White	423 (90.4)	404 (90.0)	827 (90.2)
*Type of admission N (%) from HES England only (n=950)*			
Elective	40 (8.3)	28 (6)	68 (7.2)
Emergency	443 (91.7)	439 (94.0)	882 (92.8)
*Type-AKI N (%) from AKI MPI + HES England only (n=950)*			
Community acquired AKI	334 (69.2)	351 (75.2)	685 (72.1)
Hospital acquired AKI	149 (30.8)	116 (24.8)	265 (27.9)
*Cause of AKI N (%)*			
Hypovolaemia	151 (30.3)	115 (23.4)	266 (26.9)
Circulatory failure	51 (10.2)	59 (12.0)	110 (11.1)
Sepsis	166 (33.3)	149 (30.3)	315 (31.8)
Rhabdomyolysis	3 (0.6)	2 (0.4)	5 (0.5)
Medication induced	16 (3.2)	24 (4.9)	40 (4.0)
Obstruction	32 (6.4)	68 (13.8)	100 (10.1)
Multifactorial	17 (3.4)	34 (6.9)	51 (5.1)
No specific cause	56 (11.2)	36 (7.3)	92 (9.3)
Not completed	6 (1.2)	4 (0.8)	10 (1.0)
*Admitting specialty N (%)*			
Medicine	350 (70.3)	364 (74.1)	714 (72.1)
Surgery	109 (21.9)	86 (17.5)	195 (19.7)
Intensive treatment unit	24 (4.8)	30 (6.1)	54 (5.4)
Discharged from emergency department	3 (0.6)	1 (0.2)	4 (0.4)
Other	11 (2.2)	10 (2.0)	2 (21.0)
Not completed	1 (0.2)	0 (0.0)	1 (0.1)
Serum creatinine re-checked within 30 days N (%) if alive 30 days post discharge (AKI stage 2 N=330, AKI stage 3 N=298, All AKI N=628)	221 (67.0)	224 (75.2)	445 (70.9)
Complications of AKI N (%) (missing N=17)			
Hyperkalaemia	59 (11.9)	130 (26.5)	189 (19.2)
Uraemia	61 (12.2)	114 (23.3)	175 (17.7
Pulmonary oedema	51 (10.3)	52 (10.6)	103 (10.5)
Acidosis	134 (27.2)	204 (41.8)	338 (34.5)
Median length of stay (days) (IQR)	10(5–19)	11(5–20)	10(5–20)
Readmission within 90 days N (%) if alive 90 days post discharge (AKI stage 2 N=304, AKI stage 3 N=283, All AKI N=587)	99 (32.6)	115 (40.6)	214 (36.5)
Renal function returned to baseline N (%)	286 (57.4)	209 (42.6)	495 (50.1)
In hospital mortality N (%)	141 (28.3)	166 (33.8)	307 (31.0)
30-day mortality N (%)	153 (30.7)	174 (35.4)	327 (33.1)
90-day mortality N (%)	186 (37.3)	202 (41.1)	388 (39.2)
1-year mortality N (%)	236 (47.4)	251 (51.1)	487 (49.2)

The patient characteristics and outcomes for patients included in this audit are in keeping with the characteristics and outcomes for patients admitted to all acute hospitals in England from December 2018 and February 2019 (supplemental table S1), suggesting patients included in this audit are a representative sample.

### Attainment of care quality indicators

Overall, AKI interventions were completed in more than 80% of episodes. Attainment of care quality indicators are shown in Table 2. Timely Consultant review (6 h) was achieved in 58.9% of all admissions. This was different when patients were admitted under different specialties, with Consultant review within 6 h achieved in 59.7% of patients admitted under medicine, 46.1% for patients admitted under surgical teams and 82.9% for patients admitted to the emergency department or intensive treatment units. Recording of urinalysis was low (41.9%), but timely interventions including antibiotics, IV fluids, diuretics, bladder catheterisation and nephrostomy/stenting was high. Whilst overall timely imaging of the renal tract was low (37.2%) it was completed within 24 h in 74% of patients who had obstruction as the cause of their AKI. Correspondence on discharge summaries to primary care physician relating to the episode of AKI was variable. The episode of AKI was mentioned on 79.3% of discharge summaries, but mention of an AKI episode where renal function had not fully recovered or advice about further blood tests, or medication reviews (when indicated) were only mentioned 61.9% and 65.8% of the time respectively.

**Table 2:** Attainment of care quality indicators from audit measures by peak stage AKI and for all episodes of AKI.

Variables	AKI stage 2	AKI stage 3	All AKI (AKI stage 2 and 3)
Total N (%)	498 (50.3)	491 (49.6)	989 (100.0)
*Clinical assessment: Timely reviews N (%)*			
Consultant review within 6 h	283 (58.0)	288 (59.8)	571 (58.9)
Medication review (dose adjustments and discontinuation within 6 h)	392 (79.5)	400 (82.1)	792 (80.8)
Fluid balance assessment within 6 h	425 (86.2)	425 (87.5)	850 (86.8)
Urinalysis test recorded	173 (35.5)	233 (48.3)	406 (41.9)
Imaging of renal tract (within 24 or within 6 h if suspected pyonephrosis)	139 (27.9)	228 (46.5)	367 (37.2)
Blood gas and acid bas recorded	328 (66.3)	372 (76.2)	700 (71.2)
*Clinical management: Timely interventions N (%)*			
Antibiotics within 1 h (indicated AKI stage 2 N=302, AKI stage 3 N=310)	274 (90.7)	271 (87.4)	545 (89.1)
IV fluids (indicated AKI stage 2 N=400, AKI stage 3 N=398)	384 (96.0)	391 (98.2)	775 (97.1)
Diuretics (indicated AKI stage 2 N=47, AKI stage 3 N=46)	47 (100.0)	45 (97.8)	92 (98.9)
Bladder catheterisation (indicated AKI stage 2 N=226, AKI stage 3 N=299)	210 (92.9)	281 (94.0)	491 (93.5)
Nephrostomy/Stent (indicated AKI stage 2 N=14, AKI stage 3 N=38)	13 (92.9)	33 (86.8)	46 (88.5)
*Follow-up and primary care communication N (%)*			
AKI mentioned on discharge letter (alive at discharge, AKI stage 2 N=357, AKI stage 3 N=325)	250 (71.7)	285 (87.7)	541 (79.3)
Information on discharge letter to GP re medicine changes/review or recommended blood tests when applicable (indicated AKI stage 2 N=274, AKI stage 3 279)	177 (64.6)	187 (67.0)	364 (65.8)
Follow-up of unresolved renal function mentioned on discharge letter (indicated AKI stage 2 169, AKI stage 3 201)	98 (58.0)	131 (65.2)	229 (61.9)

### Associations between attainment of care quality indicators and mortality

The univariate associations (adjusted for age) between attainment of care quality indicators and 30-day, 90-day and 1-year mortality are shown in supplemental table S2. For quality indicators relating to the assessment of patients with AKI, having a urinalysis test recorded in the notes and timely imaging of the renal tract were strongly associated with a reduced odds ratio for 30-day, 90-day and 1-year mortality. Having had a blood gas and acid base status recorded in the notes associated strongly with an increased odds ratio for 30-day, 90-day and 1-year mortality. None of the quality indicators relating to clinical management associated with mortality. Relating to patient follow-up from discharge summaries, both instructions relating to medication changes/re-commencement and follow-up blood tests and specific follow-up required for the monitoring or management of patients whose renal function had not returned to baseline were strongly associated with reduced odds ratio for mortality at 30-days, 90-days and 1-year.

On multivariable analysis of factors associated with 30-day mortality there were 327 deaths amongst 950 people. The model was adjusted for age, comorbidity, AKI stage and AKI cause, in addition to two factors felt clinically important (sex and admission type). Ethnicity, IMD, baseline creatinine and admission speciality were tested, but had no significant effect and were not included. Urinalysis (OR 0.52, CI 0.38–0.71) and undergoing an ultrasound (OR 0.68 CI 0.49–0.94) were both associated with odds of lower mortality, whilst have a blood gas measurement recorded (OR 1.64, CI 1.15–2.34) a higher mortality. Results are shown in Table 3.

**Table 3:** Multivariable logistic regression adjusted by age, sex, comorbidity score, cause of AKI, stage of AKI and admission type for odd ratio of 30-days mortality by attainment of care quality indicators.

	OR	95% CI		p-value
Clinical Assessment: timely review (Yes vs No)*				
Consultant review within 6 h	1.13	0.83	1.55	0.43
Medication review (dose adjustments and discontinuation within 6 h)	0.95	0.65	1.39	0.80
Fluid balance assessment (Fluid balance assessment within 6 h)	1.16	0.74	1.81	0.51
Urinalysis test recorded	0.52	0.38	0.71	<0.0001
USS renal tract [within 24 hrs (<6 h if pyelo) or any other imaging to exclude obstruction]	0.68	0.49	0.94	0.020
Blood/gas acid-base recorded	1.64	1.15	2.34	0.006
**Clinical management: timely Interventions - treatment completed (Y vs N) when indicated**⁎⁎				
Antibiotics (Within 1 h)	0.73	0.41	1.28	0.26
IV fluids	1.17	0.42	3.22	0.77
Bladder catheterisation	1.74	0.73	4.16	0.21
**Follow-up: Discharge letter - TTO - if alive at discharge (Y vs N)**⁎⁎⁎				
AKI mentioned on discharge letter	0.73	0.27	2.01	0.54
GP Instructions re medicine and blood tests on discharge letter	0.98	0.38	2.55	0.96
Follow-up of unresolved renal function mentioned on discharge letter	0.57	0.19	1.70	0.31

⁎ some people not included because of missing data on the variable of interest (minimum cohort n=931)

⁎⁎ people excluded mainly because ‘not-indicated’ (minimum cohort n=506)

⁎⁎⁎ people excluded if died in hospital or if ‘not-indicated’ (minimum cohort n=340)

Overall 602 people were alive 30 days after discharged from hospital following AKI. Of these 150 (24.9%) had an emergency readmission to an acute hospital in 30 days. In a multivariable analysis only comorbidity (significant) and sex (borderline) were included in model adjustments. Neither mention of AKI, GP instruction on medication, nor follow-up of unresolved kidney function had any impact on the odds of readmission.

### Regional variation in care attainment indicators and mortality outcomes

**Fig. 2:**
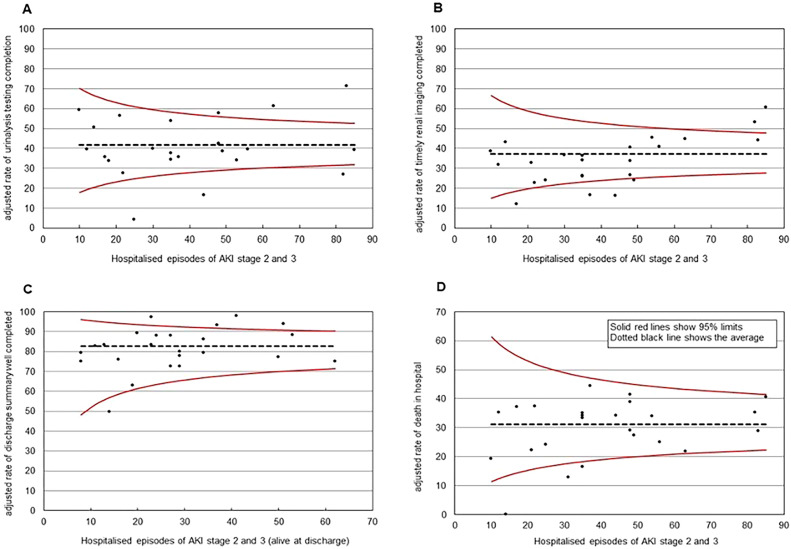
Regional variation for A: Completion of urinalysis testing, B: Timely completion of imaging of the renal tract, C: Appropriate completion of discharge summaries with information relating to AKI episode and follow-up plans, advise and instruction, and D: In hospital mortality. All adjusted for age and sex.

After adjustment for age and sex, inter-hospital variation remained in attainment of AKI care standards (Fig. 2). The variation in recording of urinalysis testing (range: 4–71.4%), timely completion of renal tract imaging (range: 11.9–60.5%) and completion of discharge summaries with relevant information relating to the AKI care episode with follow-up instruction and advice (range: 49.4–97.7%) are shown in Fig. 2. Inter-centre variation in-hospital mortality for patients identified as having an episode of AKI ranged from 0 to 44.3%.

## Discussion

This audit demonstrates that ten years after the 2009 NCEPOD report into management of hospitalised patients with AKI,^3^ significant variation remains in attainment of care quality indicators for patients hospitalised with AKI and audit standards are consistently not being met. In keeping with previous analyses of patient with AKI stages 2 and 3^15^ in-hospital mortality is high (approximately 30%), with very similar outcomes for patients with either stage 2 or stage 3 AKI. The patients included in this audit are representative of patients who were admitted with, or who develop AKI stage 2 or 3 in acute Trusts across England in 2018/2019.

It is clear that despite concerted national efforts of the last 10 years, variations in the care given to patients with AKI across the UK still exist that may lead to differential outcomes. These need addressing. Whilst this report identifies potential relationships between care quality indicators and patient outcomes, the design of the work means causal links between care indicators and outcomes cannot be inferred. It seems unlikely that simply improving completion of these care quality indicators will lead to improved patient outcomes without appropriate education, training and standardisation of processes. Indeed, many of the care quality indicators are well established, NICE recommendations of best practice,^9^ and whilst it is disappointing, for example, that in a condition with a 30% mortality only 60% of patients received the best practice review by a Consultant within 6 h, simply improving completion of this care quality indicator alone is unlikely to improve outcomes.

These data identify discharge from hospital as a key point in the transition of care that may influence outcomes. The finding that discharge correspondence to primary care that included information on further tests, follow-up or medication optimisation associated with improved mortality is novel. Given the 90-day readmission rate following an episode of AKI amongst survivors was 30–40% it is perhaps surprising that on multivariable analyses the effect of recording of these factors on discharge summaries on readmission rates was lost. Given that 20% of all discharge summaries did not even include mention of the episode of AKI, this is perhaps an area where concerted efforts at improvements could be made.

This audit has identified care quality indicators that may have a relationship with patient mortality, though care is needed in the interpretation of the findings. Completion of urinalysis and timely imaging of the urinary tract associated with patient mortality on both univariate and multivariate analyses. However, the majority of cases of AKI were attributed to pre-renal causes (e.g. sepsis, hypovalaemia), so early ultrasound scan and urinalysis were unlikely to have been considered priorities in the care of these potentially very ill patients and the statistical relationship with mortality is likely to be explained by confounding. Indeed, it is difficult to see how delay in many of the investigations could have contributed to mortality in most cases as they would not have altered the initial management of patients with AKI caused by sepsis or hypovolaemia. No care quality indicators of the initial clinical management of patients with AKI (including appropriate administration or IV fluids, antibiotics, diuretics, bladder catheters or nephrostomies/stents) associated with patient mortality.

### Strengths and limitations

This was a large national audit with HES linkage to 30-day, 90-day and 1-year mortality. The NEPHwork AKI audit differed from the NCEPOD audit because it included survivors of hospital admissions including an episode of AKI whilst the NECPOD audit only reviewed the care of those who had died. The proportion of people who achieved each care standard cannot therefore be directly compared. Additionally, cases identified in the NEPHwork AKI audit using the nationally mandated AKI warning test score were significantly more likely to have clinical AKI (989 out of 1,187 episodes) than those identified through clinical coding in the previous NCEPOD audit supporting the findings that the warning algorithm is specific.^16^ The differing case selection method does mean, however, that no comment is possible on any cases of clinical AKI not detected. Whilst this audit does provide novel data, as discussed data are entirely observational. It is not possible to adjust for all possible co-variates and residual confounding will account for many of the relationships described. For example, whilst imaging of the renal tract and recording of urinalysis associated with mortality in multivariable models, despite adjusting for cause and stage of AKI, the reduced mortality observed is likely to be explained by confounding by indication bias. Moreover, whilst analyses were adjusted for cause of AKI and co-morbidity, we could not adjust for severity of acute illness as this information was not available. As demonstrated by the relationship between documented blood gas/acid base and increased mortality, a causal relationship is extremely unlikely and likely reflects the fact that patients felt to be more unwell were more likely to have a blood gas. Causality cannot be inferred from these data and must not be over-interpreted.

## Conclusions

This National audit of AKI in acute hospital trusts across England and Wales shows that care and attainment of care quality indicators remains variable and outcomes remain poor. Simply improving attainment of care quality indicators alone is unlikely to improve patient outcomes. Discharge may be a key transition point to explore in future research and quality improvement projects to develop and test interventions to improve standardisation of care and outcomes.

## Funding statement

This work was supported by a special project grant from Kidney Research UK (NEPHWork Project One) and the UK Kidney Association

## Author contributions

MGB led project design, supported delivery, interpretation of results, prepared the manuscript draft, oversaw manuscript revisions and finalisation. AC supported project management, completed statistical analyses, was involved in interpretation of results manuscript revision and finalisation. MS was involved in project design and delivery, led project management, supported and oversaw audit setup, data collection and queries, statistical analyses, interpretation of results manuscript revision and finalisation. TH was involved in project design and delivery, interpretation of results and manuscript revisions. RP was involved in project design and delivery, interpretation of results and manuscript revisions. MA was involved in project delivery, interpretation of results and manuscript revisions. JW was involved in project delivery, interpretation of results and manuscript revisions. KC was involved in project delivery, interpretation of results and manuscript revisions. JFM supported project design, oversaw governance relating to data handling through the UKRR, interpretation of results, manuscript revisions and finalisation

## Declaration of Competing Interest

JFM, AS, MS and RP are or were funded by the UK Renal Registry who supported development and delivery of this work. JW – previously funded by Kidney Research UK. TH, MGB, JW, KC – nil competing interests

## Appendix Supplementary materials

Image, application 1

## References

[1] UK Renal Registry (2020) Acute kidney injury (AKI) in England – a report on the nationwide collection of AKI warning test scores from 2018.

[2] Holmes J., Rainer T., Geen J. (2016). Acute kidney injury in the era of the AKI e-alert. Clin J Am Soc Nephrol.

[3] NCEPOD (2009). Acute Kidney Injury: Adding Insult to Injury (2009). Available from: ncepod.org.uk/2009akitoolkit.html.

[4] Lameire N.H., Bagga A., Cruz D. (2013). Acute kidney injury: an increasing global concern. Lancet.

[5] Selby N.M., Fluck R.J., Kolhe N.V. (2016). International criteria for acute kidney injury: advantages and remaining challenges. PLoS Med.

[6] Savino M, Plumb L, Casula A (2021). Acute kidney injury identification for pharmacoepidemiologic studies: Use of laboratory electronic acute kidney injury alerts versus electronic health records in Hospital Episode Statistics. Pharmacoepidemiol Drug Saf.

[7] UK Renal Registry (2020) Acute kidney injury (AKI) in England – a report on the nationwide collection of AKI warning test scores from 2018.

[8] Crowe K, Savino M, Williams J, Amaran M, Humphrey T, Medcalf J (2022). NEPHwork consortium. NEPHwork: creating a quality improvement and research network for UK renal trainees. BMJ Open Qual.

[9] NICE Guideline: Emergency and acute medical care in over 16s. Quality standard [QS174], Published: 07 September 2018. (https://www.nice.org.uk/guidance/qs174 Accessed July 2023).

[10] UK Renal Association Clinical Practice Guideline, Acute Kidney Injury (AKI), 2019. (https://ukkidney.org/sites/renal.org/files/FINAL-AKI-Guideline.pdf Accessed July 2023).

[11] NHS England (2015) Acute kidney injury (AKI) algorithm. (england.nhs.uk/akiprogramme/aki-algorithm. Accessed July 2023).

[12] Kidney Disease: Improving Global Outcomes (KDIGO) (2012) KDIGO clinical practice guideline for acute kidney injury. Kidney Int. 2 (suppl. 1): 1–138.

[13] GOV.UK (2015). English indices of deprivation 2015. (gov.uk/government/statistics/english-indices-of-deprivation-2015. Accessed July 2023).

[14] Peracha Javeria, Pitcher David, Santhakumaran Shalini (2022). Centre variation in mortality following post-hospitalization acute kidney injury: analysis of a large national cohort. Nephrol Dialysis Transp.

[15] Selby NM (2019). An organizational-level program of intervention for AKI: a pragmatic stepped wedge cluster randomized trial. J Am Soc Nephrol.

[16] Sawhney S (2015). Maximising Acute Kidney Injury Alerts – a cross-sectional comparison with the clinical diagnosis. PLoS One.

