# Supplementary material for: A National audit of the care of patients with acute kidney injury in England and Wales in 2019 and the association with patient outcomes
Source: Clin Med (Lond). 2024 Feb 20;24(2):100028. doi: 10.1016/j.clinme.2024.100028 (PMC11091442; doi:10.1016/j.clinme.2024.100028)
Supplement: Supplementary file 1 [file mmc1.docx]

# Appendix:

Supplemental table S1: Characteristics and outcomes of patients with stage 2 or 3 AKI included in this audit, compared to the characteristics and outcomes of all patients with AKI stage 2 or 3 admitted to acute hospital Trusts in England from December 2018 to February 2019. Data obtained from the UK Renal Registry.

| **Variables** | **Patients with AKI stage 2 or 3 included in NEPHwork Audit** | **Patients with AKI stage 2 or 3 in acute Hospital Trusts across England from December 2018 to February 2019** |
| --- | --- | --- |
| Total (n) | 989 | 34421 |
| *AKI stage (%)* | | |
| aki-2 | 50.3 | 55.1 |
| aki-3 | 49.6 | 44.9 |
| *Age group (%)* | | |
| 18-29 | 1.7 | 1.8 |
| 30-49 | 7.8 | 7.7 |
| 50-64 | 16.9 | 17.7 |
| 65-74 | 21.0 | 22.8 |
| 75-84 | 30.1 | 27.8 |
| ≥85 | 22.3 | 22.3 |
| Sex (%) | |  |
| Female | 45.9 | 46.9 |
| Male | 54.1 | 53.1 |
| *Index of Multiple Deprivation quintile (%)* | | |
| Deprivation score-1 | 18.8 | 16.1 |
| Deprivation score-2 | 22.0 | 19 |
| Deprivation score-3 | 20.5 | 19.4 |
| Deprivation score-4 | 19.1 | 21.1 |
| Deprivation score-5 | 19.7 | 24.3 |
| *Type of admission (%)* | | |
| Elective | 7.2 | 6.1 |
| Emergency | 92.8 | 93.9 |
| *Type-AKI N (%)* | | |
| Community acquired AKI | 72.1 | 69.8 |
| Hospital acquired AKI | 27.9 | 30.2 |
| *Mortality outcomes (%)* | | |
| In hospital mortality | 31.0 | 28.1 |
| 30-day mortality | 33.1 | 32.4 |
| 90-day mortality | 39.2 | 40.8 |
| 1-year mortality | 49.2 | 51.1 |

Table S2: Univariate logistic regression adjusted by age for odd ratio of mortality by attainment of care quality indicators

|  | **30-days mortality (327 events)** | | | | **90-days mortality (388 events)** | | | | **1-yr mortality (487 events)** | | | |
| --- | --- | --- | --- | --- | --- | --- | --- | --- | --- | --- | --- | --- |
|  | **ALL STAGES** | | | | **ALL STAGES** | | | | **ALL STAGES** | | | |
|  | **OR** | **95% CI** |  | **p-value** | **OR** | **95% CI** |  | **p-value** | **OR** | **95% CI** |  | **p-value** |
| **Clinical Assessment: timely review (Yes vs No)** |  |  |  |  |  |  |  |  |  |  |  |  |
| Consultant review within 6 hours | 1.3 | 0.98 | 1.72 | 0.066 | 1.16 | 0.89 | 1.53 | 0.27 | 1.37 | 1.05 | 1.79 | 0.022 |
| Medication review (dose adjustments and discontinuation within 6 hours) | 1.08 | 0.77 | 1.53 | 0.65 | 1.04 | 0.74 | 1.46 | 0.82 | 0.96 | 0.69 | 1.34 | 0.82 |
| Fluid balance assessment (Fluid balance assessment within 6 hours) | 1.28 | 0.85 | 1.93 | 0.25 | 1.08 | 0.73 | 1.6 | 0.69 | 1.17 | 0.8 | 1.71 | 0.43 |
| Urinalysis test recorded | 0.54 | 0.41 | 0.72 | <0.0001 | 0.58 | 0.44 | 0.76 | 0.0001 | 0.59 | 0.45 | 0.77 | 0.0001 |
| USS renal tract [within 24hrs (<6hrs if pyelo) or any other imaging to exclude obstruction] | 0.64 | 0.48 | 0.85 | 0.0024 | 0.69 | 0.52 | 0.91 | 0.0075 | 0.73 | 0.56 | 0.96 | 0.023 |
| Blood/gas acid-base recorded | 1.83 | 1.33 | 2.51 | 0.0002 | 1.75 | 1.29 | 2.36 | 0.0003 | 1.58 | 1.19 | 2.12 | 0.002 |
| **Clinical management: timely Interventions - treatment completed when indicated** |  |  |  |  |  |  |  |  |  |  |  |  |
| Antibiotics (Within 1 hour) (indicated, with data, N=612, 302 AKI stage 2 - 310 AKI stage 3) | 0.898 | 0.529 | 1.526 | 0.69 | 0.92 | 0.55 | 1.56 | 0.77 | 0.8 | 0.47 | 1.37 | 0.42 |
| IV fluids (indicated, with data, N=798, 400 AKI stage 2 - 398 AKI stage 3) | 1.262 | 0.483 | 3.3 | 0.63 | 1.12 | 0.46 | 2.75 | 0.8 | 1.05 | 0.44 | 2.49 | 0.91 |
| Bladder catheterization (indicated, with data, N=525, 226 AKI stage 2 - 299 AKI stage 3) | 1.481 | 0.669 | 3.278 | 0.33 | 1.94 | 0.88 | 4.3 | 0.1 | 1.11 | 0.54 | 2.27 | 0.78 |
| **Follow-up: Discharge letter - TTO** |  |  |  |  |  |  |  |  |  |  |  |  |
| AKI mentioned on discharge letter (alive at discharge, N= 682, 357 AKI stage 2 - 325 AKI stage 3) | 0.83 | 0.35 | 2 | 0.68 | 0.92 | 0.5 | 1.7 | 0.79 | 1.03 | 0.65 | 1.62 | 0.9 |
| GP Instructions re medicine and blood tests on discharge letter when applicable (with data N = 553, 274 AKI stage 2 - 279 AKI stage 3) | 0.26 | 0.14 | 0.51 | <0.0001 | 0.52 | 0.31 | 0.87 | 0.012 | 0.65 | 0.43 | 0.96 | 0.032 |
| Follow-up of unresolved renal function mentioned on discharge letter (applicable, with data, N= 370, 169 AKI stage 2 - 201 AKI stage 3) | 0.16 | 0.07 | 0.36 | <0.0001 | 0.32 | 0.18 | 0.57 | 0.0001 | 0.56 | 0.35 | 0.88 | 0.013 |
